# Double‐Blind, Randomized, Placebo‐Controlled Trial of DA‐9701 in Parkinson's Disease: PASS‐GI Study

**DOI:** 10.1002/mds.28219

**Published:** 2020-08-06

**Authors:** Ji‐Hyun Choi, Jee‐Young Lee, Jin Whan Cho, Seong‐Beom Koh, Young Soon Yang, Dalla Yoo, Cheol‐Min Shin, Hee Tae Kim

**Affiliations:** ^1^ Department of Neurology, Seoul Metropolitan Government‐Seoul National University Boramae Medical Center Seoul National University College of Medicine Seoul South Korea; ^2^ Department of Neurology, Samsung Medical Center Sungkyunkwan University School of Medicine Seoul South Korea; ^3^ Department of Neurology Korea University Guro Hospital Seoul South Korea; ^4^ Department of Neurology National Neuroscience Institute Singapore Singapore; ^5^ Department of Neurology Kyung Hee University Hospital Seoul South Korea; ^6^ Division of Gastroenterology, Department of Internal Medicine Seoul National University Bundang Hospital Seongnam South Korea; ^7^ Department of Neurology Hanyang University Medical Center Seoul South Korea

**Keywords:** DA‐9701, double‐blind randomized controlled trial, gastrointestinal dysfunction, Parkinson's disease, quality of life

## Abstract

**Objectives:**

This study aimed to assess the efficacy of DA‐9701 on gastrointestinal symptom‐related quality of life in patients with Parkinson's disease on stable dopaminergic medications.

**Methods:**

This multicenter, double‐blind, placebo‐controlled, phase 4 trial included a total of 144 patients with Parkinson's disease with gastrointestinal dysfunctions based on predefined criteria. Participants were randomized to take either DA‐9701 or placebo for 4 weeks, and then both groups were administered DA‐9701 for an additional 8 weeks while antiparkinsonian medications were unchanged. The primary outcome measure was gastrointestinal symptoms and related quality‐of‐life changes assessed on the Korean Nepean dyspepsia index after 4 and 12 weeks of therapy. We also evaluated the impact of DA‐9701 therapy on parkinsonian motor symptoms at each time point.

**Results:**

The gastrointestinal symptom‐related quality‐of‐life score significantly improved in the DA‐9701‐treated group compared with the placebo‐treated group after 4weeks (adjusted *P* = 0.012 by linear mixed effect model analysis). The overall gastrointestinal symptom and dyspepsia sum scores improved at 12 weeks after intervention in the DA‐9701‐first treated group (adjusted *P* = 0.002 and 0.014, respectively) and also in the placebo‐first treated group (adjusted *P* = 0.019 and 0.039) compared with the baseline. Parkinsonian motor severity was not significantly affected by DA‐9701 treatment in both groups at 4 and 12 weeks after intervention. There were no drug‐related serious adverse events throughout the trial.

**Conclusions:**

DA‐9701 therapy improved gastrointestinal symptom‐related quality of life, and 12 weeks of daily administration can relieve the overall severity of gastrointestinal symptoms in patients with Parkinson's disease without affecting motor symptoms. (Clinical trial identifier: NCT02775591.) © 2020 The Authors. *Movement Disorders* published by Wiley Periodicals LLC on behalf of International Parkinson and Movement Disorder Society.

Gastrointestinal (GI) dysfunction is a common nonmotor feature of Parkinson's disease (PD) affecting approximately two thirds of patients with PD.^1^, ^2^ Dysphagia, nausea/vomiting, early satiety, bloating, abdominal distension, and constipation are major complaints of patients, which are related to GI dysmotility.^2^ Pathological involvement of vagal motor nucleus, peripheral α‐synucleinopathies, and the effect of oral antiparkinsonian medications interactively contribute to the high prevalence of GI dysfunction in PD.^3^, ^4^, ^5^, ^6^

The prokinetics commercially available have primary targets of 5‐hydroxytryptamine type 4 (5‐HT_4_) and dopamine type 2 (D_2_) receptors expressed in the GI tract.^7^ Agonizing other types of receptors such as 5‐HT_1A,_ 5‐HT_1B/D_, and 5‐HT_3,_ adrenergic α_2_, muscarinic, and opioid receptors can reportedly enhance gastric accommodation and reduce visceral pain in humans.^8^, ^9^, ^10^, ^11^, ^12^, ^13^ DA‐9701 is a recently developed prokinetic derived from Corydalis Tuber and Pharbitis Semen (Motilitone, Dong‐A ST, Seoul, Republic of Korea).^7^ It has been marketed in Korea since 2011 after the Korea Food and Drug Administration's approval for the treatment of functional dyspepsia related to the upper GI dysfunction. By the unique properties of its composite,^14^ DA‐9701 has been demonstrated to have an agonistic effect on 5‐HT_1A,_ 5‐HT_4_, and α_2_ receptors, and antagonistic effect on D_2_ receptor in the GI tract.^15^ In in vivo studies, DA‐9701 enhances gastric motility and accommodation as well as reduces visceral hypersensitivity.^16^, ^17^, ^18^, ^19^, ^20^, ^21^

There are several clinical trials with DA‐9701 conducted in the non‐PD population. In patients with functional dyspepsia, DA‐9701 showed an efficacy noninferior to itopride (another D_2_ antagonistic prokinetic).^22^ A dynamic magnetic resonance imaging study revealed that DA‐9701 enhances gastric emptying in healthy elderly populations.^23^ In people with chronic constipation, DA‐9701 improved colonic motility revealed by colon transit time.^24^ A recent small‐scale, head‐to‐head trial has shown noninferior efficacy of DA‐9701 over domperidone on gastric dysmotility symptoms in patients with PD.^25^ This study provided real‐time dynamic magnetic resonance imaging measures demonstrating the gastrokinetic effect of DA‐9701 resulting in improving gastric emptying.^25^ Of note, DA‐9701 did not induce hyperprolactinemia in all participating subjects in contrast to domperidone.^25^

Although pieces of evidence have shown the possible benefit of DA‐9701 on enhancing GI motility, there has been no randomized controlled trial of DA‐9701 on the symptom‐related quality of life (QoL) in patients with PD with GI disturbance. We needed to investigate whether the daily administration of DA‐9701 affects parkinsonian symptoms in patients with PD on concomitant dopaminergic medications, considering the safety concern regarding its GI D2 antagonizing effect. Therefore, we conducted a multicenter, double‐blind, randomized, placebo‐controlled trial of DA‐9701 in patients with PD on stable dopaminergic medications.

## Methods

### Study Subjects

The protocol of this trial was registered to ClinicalTrial.gov (NCT02775591). The study participants were recruited from 5 referral hospitals in Seoul, South Korea, between April 2016 and March 2018. The inclusion criteria were patients with PD diagnosed according to the UK PD Brain Bank diagnostic criteria^26^ and on stable antiparkinsonian medications for at least 3 months. The patients must be aged between 50 and 80 years and have 1 or more of the following GI symptoms: anorexia, nausea, vomiting, early satiety, symptoms of gastroesophageal reflux, abdominal distension or pain, constipation, and defecation problems. We excluded patients from this study if they had cognitive impairment with the Mini‐Mental State Examination score < 20, significant motor complications with fluctuations or dyskinesias, history of abdominal surgery, active GI diseases requiring gastroenterology treatment within 1 month prior to the baseline, use of any prokinetics (ie, mosapride, itopride, domperidone, motilitone, levosulpiride, metoclopramide, prucalopride) within 2 months prior to this study, severe comorbidities that could interfere with QoL or daily activities of patients, history of hypersensitivity or adverse events related to DA‐9701 use, or participation in other clinical trials within 3 months prior to the baseline. Patients on chronic use of antipsychotics or antidepressants were also excluded, but users of stable dose benzodiazepines or sleep pills could be included if they were kept in fixed dose during the participation in this trial. The institutional review boards of 5 participating centers approved this study. Every participant provided written informed consent before entering the trial.

### Randomization and Masking

Randomization was performed at baseline visit according to a predesigned randomization table made by a consultant statistician at Seoul National University Boramae Medical Center with a stratified permuted block design^27^ considering an institution and Hoehn and Yahr stage (<2.5 or not). The investigational drugs were prepacked according to the serial number provided in the random table by an independent research pharmacist to keep triple blindness to investigators, participants, and study pharmacists who were involved in this trial.

### Sample Size Estimation

We presumed a significant difference in mean changes in the dyspepsia sum score was 12 and the standard deviation was 24 according to previous study results.^28^ The required sample size was 63 for each arm with α = 0.05 under statistical power of 80%. Assuming a 15% drop‐out rate, we estimated the sample size per group as 75.

### Procedures

After enrollment, participants were randomized to take either DA‐9701 30 mg or placebo (a dummy tablet in the same color and size with DA‐9701) 3 times a day for 4 weeks (1:1 ratio). Then both groups were administered DA‐9701 for an additional 8 weeks. We maintained double blindness until the end of this trial. Antiparkinsonian medications were unchanged throughout the trial. At baseline, we obtained demographics and clinical information, including PD duration, daily levodopa dose, and levodopa equivalent daily dose.^29^ Outcome measure evaluations and adverse event monitoring were performed at baseline, 4 and 12 weeks after intervention, and on unexpected visits.

### Outcome Measures

#### GI Symptoms and QoL

We assessed GI symptoms using the Nepean Dyspepsia Index–Korean Version (NDI‐K). The NDI‐K questionnaire consista of 15 items. Participants rated each item for frequency (scored 0–4), intensity (scored 0–5), and disability (scored 0–4).^28^, ^30^, ^31^ The dyspepsia sum score in the NDI‐K is a summation of 8 cardinal items: upper abdominal pain, discomfort, burning, pressure and bloating sensation, inability to finish a regular meal, fullness after eating, and nausea. We defined the clinically relevant improvement as a 50% or more decrease in the dyspepsia sum score following the criteria used in functional dyspepsia studies.^28^, ^30^, ^31^ The second part of the NDI‐K consists of QoL questionnaire related to GI symptoms. It has the following 5 domains with 25 items: tension/sleep, interference with daily activities, eating/drinking, knowledge/control, and work/study. Each domain score range from 0 to 100 (better GI‐related QoL with higher score).^29^, ^30^, ^31^ The NDI‐K GI symptom and QoL questionnaires showed acceptable test–retest reproducibility and internal consistency in validation studies.^30^, ^31^ Furthermore, it has been widely used in trials on GI dysmotility or functional disorders.^22^, ^28^, ^31^, ^32^

#### Parkinsonian Symptoms and QoL

We assessed the effect of DA‐9701 on parkinsonian motor symptoms and PD‐related QoL by the Unified Parkinson's Disease Rating Scale (UPDRS), Hoehn and Yahr stage, and the Korean version 39‐item Parkinson's Disease Questionnaire (K‐PDQ39), consisting of 8 domains with scores ranging from 0 to 100 (worse PD‐related QoL with higher scores).^33^ Participants were evaluated at the medication *on* state.

#### GI Symptom Diary

For an explorative analysis, we adopted a GI symptom diary for 1 week before every visit in this trial. The GI symptom diary had checkpoints for frequencies of inability to finish regular meals as a result of early fullness, bloating in the upper abdomen after a meal, and pain in the upper abdomen. It also included defecation frequency and the Bristol stool scale.^34^

### Statistical Analysis

Efficacy measures were analyzed within an intention‐to‐treat paradigm. In the comparison of baseline characteristics and GI‐related parameters of the DA‐9701 and placebo groups, we used the Student's *t* test or Mann‐Whitney *U* test for continuous variables and the χ^2^ test or Fisher's exact test for categorical variables. For the primary outcomes, we employed a linear mixed‐effect model (LMM) for group comparisons of NDI‐K score changes at 4 weeks and temporal changes at 4 and 12 weeks from baseline in each group. The LMM performs statistical adjustment for baseline differences between the groups by imputing baseline scores as covariates in the model and uses all available data and provides valid results in the presence of missing data under the assumption that missing data are missing at random.^35^, ^36^ We also applied LMM for the UPDRS and the K‐PDQ39 score changes. Age, sex, and baseline scores were considered for every model as covariates. For changes in GI symptom diaries, we used the Wilcoxon signed‐rank test. All statistical analyses were performed using SPSS version 26.0 (IBM Corp., Armonk, NY) and R (version 3.5.3.; http://www.r-project.org) with a significance level set at 0.05 (2‐tailed).

### Role of the Funding Source and Data Availability Statement

The study funder had no roles in study design, data collection, analysis and interpretation, or writing of the article. Data supporting the findings of this study are available within the article and supplementary material. Upon reasonable request, additional raw data could be offered by the corresponding author.

## Results

### Participants

**FIG. 1.**
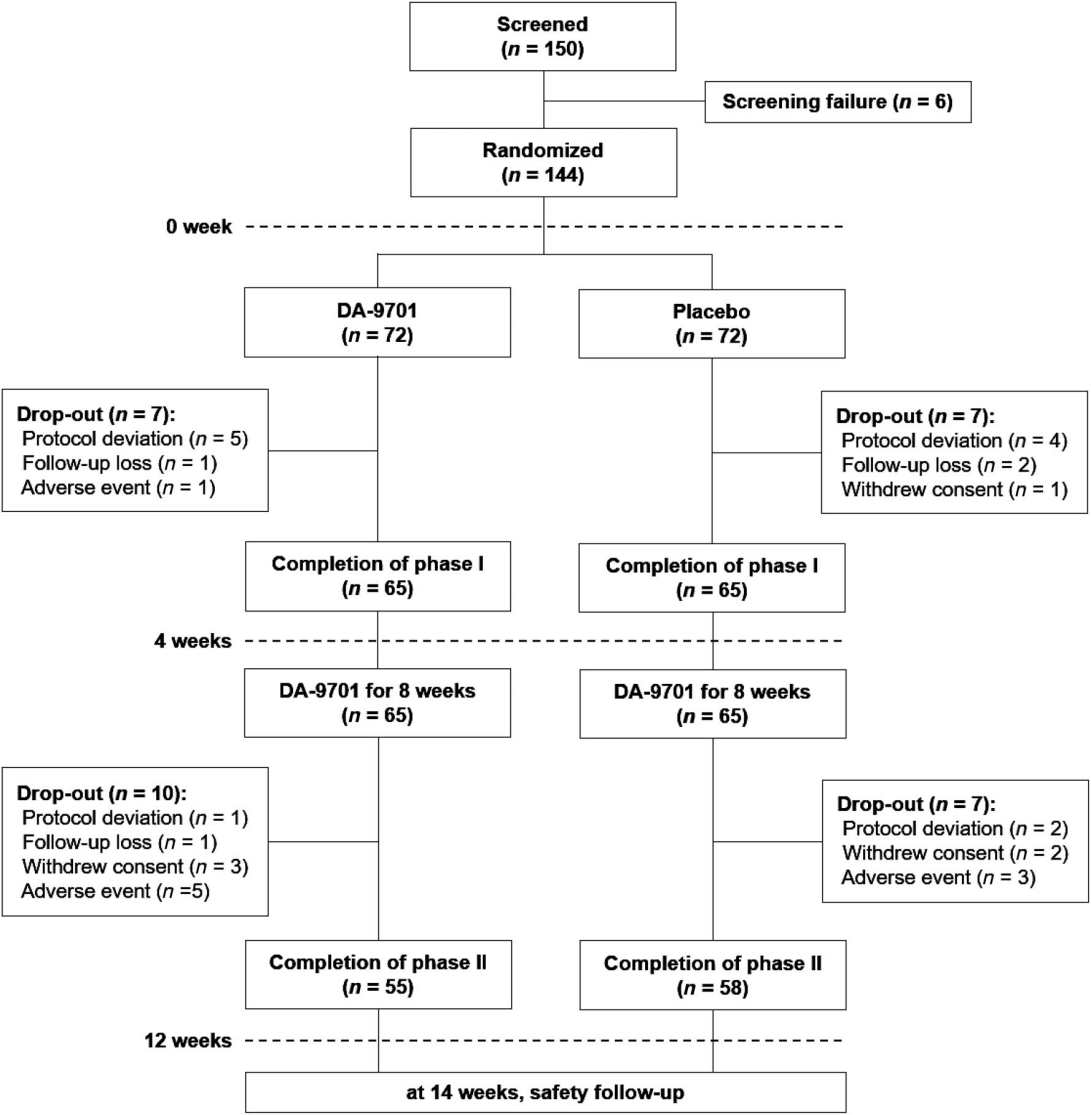
Flowchart of study subjects. We screened a total of 150 participants. Among them, 65 participants in each DA‐9701 and placebo arm (a total of 130 participants) completed the first 4 weeks’ parallel intervention; 55 participants of the DA‐9701‐first and 58 participants of the placebo‐first groups (a total of 113 participants) completed all study visits.

We screened a total of 150 participants. A total of 144 participants were randomly allocated to either DA‐9701 or the placebo group. During the first 4 and additional 8 weeks of intervention, 14 and 17 participants dropped out (Fig. 1). Therefore, a total of 113 participants completed 12 weeks of assessments in this trial.

### Baseline Characteristics of the Participants

There were no differences between the DA‐9701 and placebo groups in age and sex distribution, PD duration, dopaminergic medication doses, UPDRS scores, Hoehn and Yahr stages, and Mini‐Mental State Examination (Table 1). We provide baseline scores on the NDI‐K symptom and QoL questionnaires in DA‐9701 and the placebo groups in Supplementary Table 1. The NDI‐K symptom and QoL scores at baseline were slightly worse in the DA‐9701 group (the NDI‐K symptom score = 20.63 ± 20.19 vs. 14.51 ± 15.01, *P* = 0.042; dyspepsia sum score = 13.85 ± 13.56 vs. 9.74 ± 11.11, *P* = 0.049; and NDI‐K QoL score = 49.29 ± 20.59 vs. 57.02 ± 22.69, *P* = 0.035, for DA‐9701 vs. the placebo groups). Therefore, we adjusted baseline GI symptom scores in the primary and secondary outcome analyses using LMM. Furthermore, we also conducted the same analyses in a subset of participants with the NDI‐K symptom score > 15 (above median value) to evaluate the efficacy of DA‐9701 over placebo in a group with severe GI symptoms (*N* = 39 and 27 for each group) for whom the baseline NDI‐K symptom and QoL scores were not different between the DA‐9701 and placebo groups (*P* = 0.453 and 0.724, respectively). The 2 groups were indifferent for all items of baseline GI symptoms diaries (Supplementary Table 1).

**TABLE 1.** Baseline characteristics of the study participants

	Total randomized, n = 144	DA‐9701, n = 72	Placebo, n = 72	*P*
Age, y, mean (SD)	69.7 (7.3)	70.2 (7.5)	69.2 (7.1)	0.411^b^
Men, n (%)	78 (54.2)	42 (58.3)	36 (50.0)	0.316^c^
PD duration, mo, median (IQR)	24.0 (7.0–60.0)	26.5 (8.0–70.0)	23.0 (7.0–45.5)	0.133^d^
PD medication duration, mo, median (IQR)	24.0 (7.0–59.5)	25.5 (8.0–70.0)	21.0 (6.0–45.5)	0.091^d^
UPDRS Part I, mean (SD)	2.4 (2.0)	2.2 (1.9)	2.5 (2.2)	0.371^b^
UPDRS Part II, mean (SD)	7.4 (5.1)	7.9 (5.5)	7.0 (4.7)	0.268^b^
UPDRS Part III, mean (SD)	20.0 (9.8)	21.5 (9.9)	18.4 (9.4)	0.051^b^
UPDRS Part IV, mean (SD)	3.8 (2.8)	4.1 (2.6)	3.4 (3.0)	0.212^b^
H&Y stage, n (%)				0.427^c^
Stage 1	16 (11.1)	8 (11.1)	8 (11.1)	
Stage 1.5	20 (13.9)	6 (8.3)	14 (19.4)	
Stage 2	79 (54.9)	42 (58.3)	37 (51.4)	
Stage 2.5	16 (11.1)	8 (11.1)	8 (11.1)	
Stage 3	12 (8.3)	7 (9.7)	5 (6.9)	
Stage 4	1 (0.7)	1 (1.4)	0 (0.0)	
MMSE, mean (SD)	26.9 (2.2)	26.9 (2.0)	27.0 (2.4)	0.766^b^
Total LED, mg/d, median (IQR)	422.5 (250.0–600.0)	450.0 (300.0–652.5)	402.5 (200.0–550.0)	0.058^d^
Total levodopa dose, mg/d, median (IQR)	300.0 (200.0–520.0)	350.0 (287.5–556.3)	300.0 (200.0–450.0)	0.083^d^
Agonist LED, mg/d, median (IQR)	37.5 (0.0–120.0)	38.8 (0.0–131.3)	26.3 (0.0–112.5)	0.361^d^
NDI‐K symptom score (total), mean (SD)	17.55 (17.97)	20.63 (20.19)	14.51 (15.01)	**0.042**
Dyspepsia sum score,^a^ mean (SD)	11.78 (12.51)	13.85 (13.56)	9.74 (11.11)	**0.049**
NDI‐K QoL score	53.18 (21.94)	49.29 (20.59)	57.02 (22.69)	**0.035**
K‐PDQ39 summary index	18.46 (13.0)	19.44 (13.04)	17.53 (13.02)	0.416

^a^ Dyspepsia sum score comprises 8 items of the NDI‐K scale: upper abdominal pain, discomfort, burning, pressure and bloating sensation, inability to finish a regular meal, fullness after eating, and nausea.

*P*, comparison between the DA‐9701 and placebo groups by ^b^Student's *t* test, ^c^χ^2^ test, or ^d^Mann‐Whitney *U* test.

Bold indicates statistical significance (*P* < 0.05).

Abbreviations: SD, standard deviation; PD, Parkinson's disease; IQR, interquartile range; UPDRS, Unified Parkinson's Disease Rating Scale; H&Y, Hoehn and Yahr Scale; MMSE, Mini‐Mental State Examination; LED, levodopa equivalent dose; NDI‐K, Nepean Dyspepsia Index–Korean Version; QoL, quality of life; K‐PDQ39, Korean version 39‐item Parkinson's Disease Questionnaire.

### Efficacy: Primary Outcomes

#### GI Symptoms and Related QoL After 4 Weeks

**FIG. 2.**
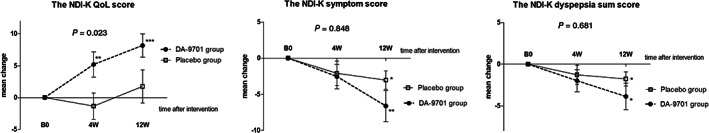
Gastrointestinal symptoms and related QoL change after intervention. The efficacy of DA‐9701 was demonstrated in the NDI‐K QoL scores at 4 weeks compared with the placebo (adjusted *P* = 0.023 by a linear mixed effect model analysis). Time changes at 4 and 12 weeks in the NDI‐K QoL scores were significant in the DA‐9701 group. There were also significant time changes in the NDI‐K total symptom scores and dyspepsia sum scores in the DA‐9701 group at 12 weeks. The placebo group also showed significant time changes in the NDI‐K symptom total score and dyspepsia scores after an additional 8 weeks of DA‐9701 therapy while maintaining double blindness until the end of the trial. *P* values provided in each figure are comparisons between DA‐9701 and the placebo groups at 4 weeks. Asterisks designate statistically significant temporal changes in the scores in each group: ^*^ < 0.05, ^**^ < 0.01, ^***^ < 0.001. NDI‐K, The Nepean Dyspepsia Index–Korean version; QoL, quality of life.

After 4 weeks of intervention, the changes in the NDI‐K symptom score and dyspepsia sum score were not different between the groups. However, the NDI‐K‐QoL score improved significantly in the DA‐9701 group compared with the placebo group (adjusted *P* = 0.023; Fig. 2).

#### GI Symptoms and Related QoL After 12 Weeks

In the DA‐9701 treatment group, the NDI‐K symptom and the dyspepsia sum scores improved significantly by further treatment for 8 weeks (adjusted *P* = 0.002 and 0.016, respectively; Fig. 2). The improvement in GI symptoms was also observed in the placebo‐first treated group in the NDI‐K symptom and the dyspepsia sum scores (adjusted *P* = 0.021 and 0.045, respectively) by an additional 8 weeks of DA‐9701 administration (Fig. 2). The NDI‐K QoL scores improved further in the DA‐9701‐treated group by 12 weeks (adjusted *P* < 0.001; Fig. 2), whereas the changes in the NDI‐K QoL in the placebo‐first treated group did not reach a significance level by 8 weeks of therapy (adjusted *P* = 0.516). The analysis in the per‐protocol set showed a similar pattern of results (Supplementary Table 2).

#### Post Hoc Analysis

A subgroup with severe GI symptoms (the NDI‐K symptom score > 15) also showed significant improvement of the NDI‐K QoL by DA‐9701 therapy in 4 weeks compared with the placebo, but not in a subgroup with mild symptoms (adjusted *P* = 0.022 and 0.215, respectively; Supplementary Table 3).

When we performed a post hoc analysis on the temporal changes in symptoms by 4 weeks, there were no items with significant changes after Bonferroni correction (Table 2). When we conducted this analysis in the per‐protocol set, the item “bitter taste fluid that comes to your mouth” showed an improvement in the DA‐9701 group (adjusted *P* = 0.002 and Bonferroni‐corrected *P* = 0.03), and there was a tendency of improvement in the nausea as well as pain and burning in the upper abdomen items (adjusted *P =* 0.025, 0.027, and 0.016, respectively; Supplementary Table 2), but again without significance after Bonferroni correction.

**TABLE 2.** Gastrointestinal symptoms and related QoL change after intervention

Least squares means from linear mixed effects model	DA‐9701				Placebo				*P* Values*				
	At 4 weeks		At 12 weeks		At 4 weeks		At 12 weeks		Between group	DA‐9701		Placebo	
	Mean change	SE	Mean change	SE	Mean change	SE	Mean change	SE	At 4 weeks	At 4 weeks	At 12 weeks	At 4 weeks	At 12 weeks
NDI‐K symptom scale													
Total symptom score	−2.50	1.70	−6.61	2.15	−2.05	1.68	−2.99	1.28	0.848	0.142	**0.002**	0.223	**0.021**
Dyspepsia sum score^a^	−1.95	1.33	−3.85	1.58	−1.22	1.15	−1.70	0.85	0.681	0.145	**0.016**	0.290	**0.045**
NDI‐K symptom items (post hoc analysis)													
Pain in upper abdomen	−0.83	0.34	−0.54	0.33	−0.50	0.24	−0.44	0.24	0.417	0.015	0.106	0.039	0.065
Discomfort in upper abdomen	−0.26	0.34	−0.52	0.32	−0.37	0.26	−0.40	0.23	0.805	0.452	0.107	0.166	0.089
Burning in upper abdomen	−0.49	0.22	−0.29	0.37	−0.02	0.26	0.02	0.22	0.161	0.027	0.433	0.952	0.914
Heartburn	0.37	0.25	−0.06	0.29	0.05	0.16	0.07	0.23	0.278	0.142	0.829	0.766	0.773
Cramps in upper abdomen	0.02	0.10	0.03	0.18	−0.10	0.15	0.07	0.09	0.517	0.857	0.874	0.499	0.435
Chest pain	−0.19	0.15	−0.24	0.25	−0.01	0.11	−0.03	0.23	0.346	0.202	0.345	0.921	0.906
Inability to finish regular meal	−0.38	0.34	−0.37	0.44	−0.48	0.30	−0.64	0.34	0.838	0.257	0.401	0.115	0.061
Bitter tasting fluid that comes to your mouth	−0.41	0.28	−0.87	0.28	−0.30	0.25	−0.32	0.26	0.774	0.150	**0.002**	0.242	0.215
Fullness after eating	0.01	0.39	0.00	0.40	0.19	0.28	−0.06	0.31	0.718	0.974	0.994	0.503	0.838
Pressure in upper abdomen	0.45	0.22	−0.23	0.24	0.09	0.12	0.21	0.12	0.151	0.040	0.349	0.456	0.080
Bloating in upper abdomen	0.21	0.33	−0.21	0.36	0.27	0.30	0.13	0.24	0.890	0.534	0.563	0.374	0.588
Nausea	−0.65	0.37	−1.41	0.40	−0.45	0.27	−0.51	0.21	0.663	0.081	**<0.001**	0.098	0.014
Belching	−0.39	0.26	−0.67	0.33	−0.39	0.28	−0.58	0.20	0.993	0.136	0.041	0.164	**0.004** ^**b**^
Vomiting	−0.09	0.29	−0.51	0.25	0.08	0.13	−0.14	0.09	0.607	0.770	0.044	0.537	0.112
Bad breath	0.09	0.11	−0.04	0.25	−0.15	0.25	−0.27	0.22	0.381	0.405	0.869	0.546	0.209
NDI‐K QoL scale													
Total QoL score	5.10	1.97	8.01	1.81	−1.41	2.03	1.68	2.59	**0.023**	**0.010**	**<0.001**	0.490	0.516
NDI‐K QoL domains (post hoc analysis)													
Tension/sleep	4.55	2.18	8.51	2.17	−1.60	2.01	−0.68	2.47	0.040	0.038	**<0.001**	0.427	0.785
Interference with daily activities	5.83	2.37	8.19	2.57	−0.64	2.17	2.80	2.56	0.046	0.014	**0.002**	0.769	0.274
Eating/drinking	6.52	2.66	8.25	2.85	−0.40	2.44	1.73	2.59	0.058	0.015	**0.004**	0.871	0.506
Knowledge/control	3.70	2.10	7.43	2.06	0.68	2.51	2.89	2.56	0.360	0.080	**<0.001**	0.788	0.260
Work/study	4.77	2.35	7.42	2.18	−5.02	3.71	1.58	6.72	0.026	0.043	**0.001**	0.177	0.814

* Adjusted *P* values for age, sex, and baseline gastrointestinal symptom severity scores for each item.

^a^ Dyspepsia sum score comprises of 8 items of the NDI‐K scale: upper abdominal pain, discomfort, burning, pressure and bloating sensation, inability to finish a regular meal, fullness after eating, and nausea.

^b^ Marginal significance as corrected, *P* = 0.06.

Bold indicates statistical significance. *P* < 0.05 is significant for total NDI‐K symptom score, dyspepsia score, and QoL scores. For each item score, significant *P* values are shown as bold based on the level after Bonferroni correction (15 items and 5 domains). Group and temporal changes in each score were analyzed by linear mixed effect models (see details in the text).

Abbreviations: QoL, quality of life; SE, standard error; NDI‐K, the Nepean Dyspepsia Index–Korean Version.

In the post hoc analysis on the NDI‐K QoL subdomains for temporal change by 4 weeks, there were no domains with significance after Bonferroni correction (Table 2). However, when we performed this analysis in the per‐protocol set, the significant temporal change after Bonferroni correction was demonstrated in 4 of 5 domains (only exception the tension/sleep domain) of NDI‐K QoL in the DA‐9701 group. In contrast, no items showed significant change in the placebo group (Supplementary Table 2).

When we performed a post hoc analysis on the NID‐K QoL subdomains for temporal changes by 12 weeks, all 5 domain scores showed significant improvement in the DA‐9701‐treated group (Bonferroni‐corrected *P* < 0.05 for all; Table 2), whereas there was no such significant change in the placebo‐treated group. In the analysis on the NDI‐K of each item by 12 weeks, the items “bitter tasting fluid that comes to your mouth” and “nausea” scores showed significant improvement in the DA‐9701 group (Bonferroni‐corrected *P* < 0.05 for both; Table 2), whereas a tendency of improvement was seen in the belching items in the placebo‐treated group (Bonferroni‐corrected *P* = 0.06; Table 2). A similar pattern of change was observed when we analyzed in a subgroup with severe GI symptoms (Supplementary Table 3).

#### Proportion of Clinically Relevant Improvement in Upper GI Symptoms

When we analyzed the proportion of clinically relevant improvement in the dyspepsia sum scores as defined in the Methods section, there were 21.5% of subjects in the DA‐9701 group and 12.3 % in the placebo group at 4 weeks (*P* = 0.160) and 30.8% versus 16.4% in the DA‐9701‐first versus placebo‐first treated group at 12 weeks (*P* = 0.078; Supplementary Fig. 1). There was no significant difference in the proportions between the 2 groups. However, in the total study population, including both 8‐week and 12‐week treated groups, the benefit of DA‐9701 was more evident in patients with severe GI disturbance than those with mild GI disturbance. The proportion of clinically relevant improvement was greater in patients with high baseline NDI‐K total symptom score and the dyspepsia sum score than those with low baseline scores (*P* = 0.022 and *P* = 0.002, respectively; Supplementary Fig. 1).

### Other Outcomes

#### GI Symptom Diaries

According to the patients’ GI symptoms diaries, improvements of pain severity in the upper abdomen was also observed after 4 weeks in the DA‐9701 group (*P* = 0.029), whereas there was no significant change in the placebo group (*P* = 0.216; Supplementary Table 4). Furthermore, 8 weeks’ DA‐9701 treatment in the placebo‐first treated group reduced frequencies of inability to finish regular meals as a result of early fullness (*P* = 0.031; Supplementary Table 4). However, other symptoms including defecation frequencies and the Bristol stool chart were not significantly changed in both groups.

#### Parkinsonian Symptoms and PD‐Related QoL After 4 and 12 Weeks

**FIG. 3.**
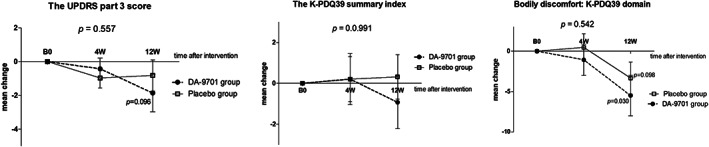
Parkinsonian symptoms and PD‐related quality of life after intervention. The UPDRS motor score and the K‐PDQ39 summary index changes were not different between DA‐9701 and the placebo groups at 4 weeks (4W). There were also no significant temporal changes in the UPDRS motor scores in both groups at 4 and 12 weeks (12W). The scores in the bodily discomfort domain of K‐PDQ39 showed a tendency of improvement at 12 weeks in the DA‐9701 group but without significance with Bonferroni correction. *P* values provided in each figure are comparisons between DA‐9701 and the placebo groups at 4 weeks. Asterisks designate statistically significant temporal changes in the scores in each group. B0, baseline; PD, Parkinson's disease, K‐PDQ39, Korean version 39‐item Parkinson's Disease Questionnaire; UPDRS, Unified Parkinson's Disease Rating Scale.

There were no differences in the changes of the UPDRS Part III scores and the K‐PDQ39 summary index between the groups after 4 weeks (adjusted *P* = 0.542 and 0.991, respectively; Fig. 3, Supplementary Table 5). The temporal changes in the UPDRS Part III and the K‐PDQ39 scores within each group were also insignificant at 4 weeks (Supplementary Table 5).

At 12 weeks, the UPDRS Part III scores tended to reduce in both groups but without statistical significance (Fig. 3, Supplementary Table 5). There were no significant temporal changes in the K‐PDQ39 for both groups. In the post hoc analysis of the K‐PDQ39 subdomains, there was a tendency of improvement in the bodily discomfort domain in both groups at 12 weeks (Supplementary Table 5) without significance after Bonferroni correction.

### Adverse Events

The incidence of adverse drug reactions was 15.4% (10/65) in the DA‐9701 group and 7.7% (5/65) in the placebo groups during the first 4 weeks (*P* = 0.173). One patient in the DA‐9701 group was hospitalized as a result of tuberculosis unrelated to the investigational drug, and 1 patient developed a contusion foot injury by accident during the first 4 weeks. During the additional 8 weeks’ intervention period, 2 patients developed abdominal discomfort, 1 did intermittent orolingual dyskinesia, 1 aggravated pre‐existing aggressiveness, and 1 developed aggravation in premorbid restless leg syndrome (Supplementary Table 6). During the safety follow‐up (+2 weeks), 1 patient complained about tinnitus. There were no serious adverse drug reactions reported throughout the trial.

## Discussion

This multicenter, double‐blinded, randomized, placebo‐controlled trial showed that DA‐9701 therapy in patients with PD on stable dopaminergic medications could improve GI symptom‐related QoL within 4 weeks and relieved overall symptoms of GI disturbances in 12 weeks without aggravating parkinsonism.

Based on the results of this trial, DA‐9701 therapy seems to be most efficacious on upper abdominal pain, burning, and pressure sensations in patients with PD. With more prolonged administration for 12 weeks, this drug also tended to relieve symptoms relating to gastroesophageal reflux, nausea, vomiting, and belching symptoms, those that are common in patients with PD on dopaminergic medications. Of note, the improvement in GI‐related QoL by DA‐9701 therapy in patients with PD tended to be pronounced with more prolonged treatment (12 weeks) compared with short‐term therapy (4 weeks).

The unique feature of the present multicenter, randomized controlled trial in PD was the implication of a structured questionnaire for GI disturbance that is validated and widely used in functional GI motility disorders. When we assessed patients’ symptoms using this scale, upper GI symptoms and visceral pain were as common as constipation in PD patients, which is allegedly the most frequent GI symptom in PD.^37^, ^38^, ^39^ Commonly reported symptoms in the study participants were inability to finish regular meal (42.4%), postprandial fullness (42.4%), abdominal bloating (40.3%), belching (38.2%), bitter‐tasting from mouth (34.7%), nausea (32.7%), abdominal pressure (16.7%), vomiting (16.7%), abdominal burning (34.7%), abdominal pain or cramp (28.5% and 15.3%), and chest pain or heartburn (25% and 18.1%). The upper abdominal symptoms were as common as constipation (41.1%) and reduced bowel movement <1 per day (43.7%) in this study population.

Upper GI tract symptoms and visceral pains originate from reduced motility and accommodation and visceral hypersensitivity.^9^, ^12^, ^40^, ^41^, ^42^ Prokinetic drugs marketed so far, either serotonin receptor agonists (cisapride, mosapride, and tegaserod) or D_2_ receptor antagonists (metoclopramide, domperidone, and itopride), have limited efficacy in patients with PD and nonnegligible side effects.^43^, ^44^, ^45^, ^46^, ^47^, ^48^ Among the D2 antagonists, metoclopramide can enhance GI motility, but it penetrates the blood–brain barrier, exerting a central D2 receptor blocking effect and causing parkinsonism aggravation. Itopride causes hyperprolactinemia^14^, ^39^, ^48^ with controversial efficacy.^43^ Domperidone shows some efficacy and does not seem to have central D2 antagonistic effect in adults; however, it can cause troublesome hyperprolactinemia and may induce cardiac arrhythmia.^44^, ^49^, ^50^, ^51^ Although cisapride and tegaserod have medicinal effects of increasing gastric emptying and decreasing gastric pain,^14^, ^52^, ^53^ the use of them is restricted by adverse effects causing cardiac arrhythmias.^45^, ^46^, ^52^, ^54^ Mosapride has been reported to be safe in patients with PD, but the efficacy data are conflicting.^48^, ^53^ Motilin agonist (camicinal) and ghrelin agonist (relamorelin) are new targets under development for gastroparesis, especially in diabetic people. A recent phase 2 study conducted in patients with PD reported that camicinal administration for 7 to 9 days enhanced gastric emptying and improved the time to peak plasma concentration (T_max_) of oral levodopa taken by the patients with PD with delayed gastric emptying.^55^ The potential beneficial effect of camicinal was highlighted by an *off*‐time reduction and increase in *on*‐time in participants of this study.^55^ Large‐scale and long‐term efficacy and safety trials are needed for this drug. Similar to these 2 drugs under development, DA‐9701 has shown efficacy in improving gastric emptying in PD.^25^ It would be interesting if further studies are initiated to reveal whether the administration of DA‐9701 could reduce *off*‐time and enhance the T_max_ of oral levodopa in patients with PD with motor fluctuation and delayed gastric emptying.

DA‐9701 was developed by a new screening frame of a Korean research and development company searching for an ideal candidate exerting “multiple actions” on GI‐tract 5‐HT_4_, 5‐HT_1,_ α_2_, and D_2_ receptors.^5^ Among the metabolites of DA‐9701, tetrahydropalmatine and tetrahydroberberine have D_2_‐receptor antagonizing activity.^56^ However, in animal studies with 50 to 100 times higher doses of DA‐9701 than a therapeutic oral dose (which is a similarly high dose for humans), the maximum concentrations of the 2 constituents in the brain were much lower than the half‐maximal inhibitory concentration values for D_2_‐receptor antagonism by both of them.^56^ This means that DA‐9701 with a therapeutic dose hardly has central antidopaminergic activity and it does not significantly induce hyperprolactinemia in humans as consistently reported by several independent studies.^5^, ^21^, ^24^ This trial was focused on GI symptoms and GI health‐related QoL in patients with PD, and most participants had stable parkinsonian motor features without fluctuation. Parkinsonian symptoms measured on the medication *on*‐state in the study participants were not significantly affected by DA‐9701 therapy during the 12 weeks’ trial. However, 1 patient developed orofacial dyskinesia, suggesting it might cause an increase in levodopa concentration by improving GI motility as reported by a previous study.^25^ The overall improving tendency of K‐PDQ39 scores of bodily discomfort domain by 12 weeks might be from facilitating levodopa absorption by improving GI motility from DA‐9701, although it remains to be proven.

The results of this study need to be interpreted with caution for several reasons. First, the placebo‐treated group did not show significant improvement in the NDI‐K QoL after 8 weeks’ DA‐9701 therapy, although this group showed some trends of improvement in the NDI‐K symptom total and the dyspepsia sum scores at the same time. This discrepancy might be related to the fact that the health‐related QoL scales are usually more affected by the lessebo effect than objective measures.^57^ Participants maintained double blindness until the end of this trial, therefore the placebo group might have negative feelings on the efficacy of the investigational drug after experiencing the first 4 weeks. Alternatively, the placebo treated group did not catch up with the DA‐9701 treated group by 8 weeks for the health‐related QoL because they got worse during the first 4 weeks and showed an improving tendency during the additional 8 weeks. In contrast, the DA‐9701 treated group showed a steady increase in their QoL. Objective measures could help clarify this issue in future trials. Second, baseline GI symptoms in the DA‐9701 group were relatively worse than the placebo group in this study. To control the possible confounding effect by this baseline characteristic, we adjusted baseline GI symptom severity in the advanced statistical models assessing the outcome measures. The overall results were favored to DA‐9701 compared with the placebo. However, the therapeutic effect was pronounced in patients with severe GI symptoms and was questionable in those with mild symptoms in a subgroup analysis. Therefore, in patients with PD with only mild GI disturbances, the therapeutic benefit of DA‐9701 has not yet been proved. Third, it might be useful to screen the severity of GI symptoms in patients with PD using GI‐specific symptom scales before therapeutic interventions. The NDI‐K is a validated GI‐specific severity scale that has been used in several trials and clinic practices.^21^, ^27^, ^28^, ^29^, ^30^ However, it needs to be further validated in the PD population to quantify the severity of GI symptoms in PD. Fourth, the outcome measures were patients’ and clinicians’ scales; thus, there was a lack of objective measure of GI motility in this trial. However, previous studies demonstrated a significant gastrokinetic effect of DA‐9701 with dynamic magnetic resonance imaging,^22^, ^24^ thereby supporting the results of the present study. One previous study reported that DA‐9701 enhances colonic transit time in patients with functional constipation.^23^ However, in our patients with PD, DA‐9701 therapy was not beneficial on constipation symptoms as seen on GI symptom diaries and the Bristol stool chart. Because constipation is the most common GI symptom in PD, the usage of DA‐9701 may be limited and not justified for patients who only complain about constipation. Other new prokinetic drugs, linaclotide and prucalopride, are considered potential drugs to be investigated in PD‐related constipation, as supported by a small retrospective study in patients with PD.^58^ Lastly, we only followed up on our patients for 14 weeks. We did not provide safety data for more extended treatment and follow‐ups, which is a practically important issue in the treatment of patients with PD. Further studies on the safety monitoring of patients with PD with prolonged use of DA‐9701 are needed in the future.

The present study provides a class I evidence on the efficacy of DA‐9701 therapy on GI symptom‐related QoL in patients with PD suffering from upper GI dysmotility symptoms. Further studies on long‐term safety issues would enhance the usage of this drug in the PD population.

## Author Roles

(1) Research project: A. Conception, B. Organization, C. Execution; (2) Statistical Analysis: A. Design, B. Execution, C. Review and Critique; (3) Manuscript: A. Writing of the first draft, B. Review and Critique.

J.H.C.: 1C, 2A, 2B, 3A

J.Y.L.: 1A, 1B, 1C, 2A, 2C, 3A, 3B

J.W.C.: 1B, 1C, 2C, 3B

S.B.K.: 1B, 1C, 2C, 3B

Y.S.Y.: 1C, 2C, 3B

D.Y.: 1C, 2C, 3B

C.M.S.: 1A, 2C, 3B

H.T.K.: 1B, 1C, 2C, 3B

## Full financial disclosures for the previous 12 months

J.Y.L. has received a research grant from National Research Foundation funded by the Ministry of Education, Science and Technology in Korea and a multidisciplinary research grant‐in‐aid and a public clinical research grant‐in‐aid from the Seoul Metropolitan Government Seoul National University Boramae Medical Center. J.H.C., J.W.C., S.B.K., Y.S.Y., D.Y., C.M.S., and H.T.K. have nothing to report.

## Supporting information


**Supplementary Table 1** Baseline gastrointestinal symptoms and related quality of life in study participants
**Supplementary Table 2**. Gastrointestinal symptoms and related quality of life change after intervention (Per Protocol set analysis)
**Supplementary Table 3A**. Gastrointestinal symptoms and related quality of life change after intervention in a subpopulation with the NDI‐K total score > 15
**Supplementary Table 3B**. Gastrointestinal symptoms and related quality of life change after intervention in a subpopulation with the NDI‐K total score ≤ 15
**Supplementary Table 4**. Gastrointestinal symptoms diary changes after 4 and 12 weeks
**Supplementary Table 5**. Parkinsonian symptoms and PD‐related quality of life change after intervention
**Supplementary Table 6**. Adverse drug reactions in this trial

**Supplementary figure 1 Proportion of clinically meaningful improvement after DA‐9701 therapy in study participants.** (A) The proportion of patients with clinically meaningful improvement (≥50% reduction in the score from baseline) between DA‐9701 and the placebo groups at 4 and 12 weeks of intervention. (B‐C) Comparison of proportion of clinically meaningful improvement at 12 weeks between patients with severe gastrointestinal symptoms and those with mild ones at baseline. Comparisons by the NDI‐K symptom total score > 15 or not (B) and by the dyspepsia sum score > 10 or not (C).
Abbreviations: NDI‐K = The Nepian Dyspepsia Index‐Korean version
