# Supplementary material for: Double‐Blind, Randomized, Placebo‐Controlled Trial of DA‐9701 in Parkinson's Disease: PASS‐GI Study
Source: Mov Disord. 2020 Aug 6;35(11):1966–76. doi: 10.1002/mds.28219 (PMC7754502; doi:10.1002/mds.28219)
Supplement: Supplementary file 1 — Supplementary Table 1 Baseline gastrointestinal symptoms and related quality of life in study participants Supplementary Table 2. Gastrointestinal symptoms and related quality of life change after intervention (Per Protocol set analysis) Supplementary Table 3A. Gastrointestinal symptoms and related quality of life change after intervention in a subpopulation with the NDI‐K total score > 15 Supplementary Table 3B. Gastrointestinal symptoms and related quality of life change after intervention in a subpopulation with the NDI‐K total score ≤ 15 Supplementary Table 4. Gastrointestinal symptoms diary changes after 4 and 12 weeks Supplementary Table 5. Parkinsonian symptoms and PD‐related quality of life change after intervention Supplementary Table 6. Adverse drug reactions in this trial [file MDS-35-1966-s001.docx]

**Supplementary Table 1.** **Baseline gastrointestinal symptoms and related quality of life in study participants**

|  | Total randomized  (n = 144) | DA-9701  (n = 72) | Placebo  (n = 72) | *p* |
| --- | --- | --- | --- | --- |
| NDI-K symptom scale^a^ |  |  |  |  |
| Total symptom score | 17.55 (17.97) | 20.63 (20.19) | 14.51 (15.01) | **0.042** |
| Dyspepsia sum score | 11.78 (12.51) | 13.85 (13.56) | 9.74 (11.11) | **0.049** |
| NDI-K symptom items |  |  |  |  |
| Pain in upper abdomen | 1.22 (2.49) | 1.48 (2.79) | 0.97 (2.15) | 0.226 |
| Discomfort in upper abdomen | 1.52 (2.67) | 1.76 (2.87) | 1.28 (2.45) | 0.281 |
| Burning in upper abdomen | 1.30 (2.12) | 1.51 (2.24) | 1.10 (1.99) | 0.249 |
| Heartburn | 0.59 (1.45) | 0.54 (1.61) | 0.64 (1.27) | 0.670 |
| Cramps in upper abdomen | 0.41 (1.24) | 0.49 (1.24) | 0.33 (1.23) | 0.442 |
| Chest pain | 0.70 (1.43) | 0.80 (1.68) | 0.60 (1.13) | 0.393 |
| Inability to finish regular meal | 2.02 (3.10) | 2.06 (3.01) | 1.99 (3.20) | 0.893 |
| Bitter tasting fluid that comes to your mouth | 1.41 (2.34) | 1.63 (2.37) | 1.18 (2.30) | 0.247 |
| Fullness after eating | 1.80 (2.69) | 2.14 (2.97) | 1.47 (2.36) | 0.138 |
| Pressure in upper abdomen | 0.55 (1.57) | 0.59 (1.84) | 0.51 (1.26) | 0.769 |
| Bloating in upper abdomen | 1.75 (2.61) | 2.15 (2.86) | 1.35 (2.30) | 0.065 |
| Nausea | 1.62 (2.68) | 2.15 (3.13) | 1.08 (2.02) | 0.017 |
| Belching | 1.52 (2.52) | 1.77 (2.86) | 1.26 (2.11) | 0.228 |
| Vomiting | 0.52 (1.57) | 0.76 (2.07) | 0.29 (0.78) | 0.078 |
| Bad breath | 0.63 (1.83) | 0.79 (2.03) | 0.47 (1.62) | 0.304 |
| NDI-K QoL scale^a^ |  |  |  |  |
| Total QoL score | 53.18 (21.94) | 49.29 (20.59) | 57.02 (22.69) | **0.035** |
| NDI-K QoL domains |  |  |  |  |
| Tension/Sleep | 53.79 (22.27) | 50.38 (19.93) | 57.16 (24.03) | 0.069 |
| Interference with daily activities | 54.52 (24.05) | 50.62 (23.53) | 58.35 (24.10) | 0.054 |
| Eating/Drinking | 54.32 (24.77) | 49.98 (24.83) | 58.60 (24.11) | 0.037 |
| Knowledge/Control | 49.42 (22.07) | 46.41 (21.33) | 52.39 (22.52) | 0.105 |
| Work/Study | 53.87 (29.25) | 49.06 (21.87) | 58.60 (34.54) | 0.050 |
| Patients’ GI symptoms diary^b^ |  |  |  |  |
| Inability to finish regular meal due to early fullness/day |  |  |  | 0.219 |
| Absent | 79 (56.4) | 34 (50.0) | 45 (62.5) |  |
| Once | 32 (22.9) | 17 (25.0) | 15 (20.8) |  |
| Twice | 10 (7.1) | 4 (5.9) | 6 (8.3) |  |
| Three times (every meal) | 19 (13.6) | 13 (19.1) | 6 (8.3) |  |
| Bloating in upper abdomen after meal/day |  |  |  | 0.582 |
| Absent | 87 (62.1) | 40 (58.8) | 47 (65.3) |  |
| Once | 36 (25.7) | 17 (25.0) | 19 (26.4) |  |
| Twice | 8 (5.7) | 5 (7.4) | 3 (4.2) |  |
| Three times (every meal) | 9 (6.4) | 6 (8.8) | 3 (4.2) |  |
| Burning or pain in upper abdomen/day |  |  |  | 0.615 |
| Absent | 100 (70.9) | 46 (66.7) | 54 (75.0) |  |
| Mild | 34 (24.1) | 19 (27.5) | 15 (20.8) |  |
| Moderate | 3 (2.1) | 1 (1.4) | 2 (2.8) |  |
| Severe | 3 (2.1) | 2 (2.9) | 1 (1.4) |  |
| Very severe | 1 (0.7) | 1 (1.4) | 0 (0.0) |  |
| Defecation frequency/day |  |  |  | 0.664 |
| < 1 | 59 (43.7) | 31 (47.7) | 28 (40.0) |  |
| 1 | 60 (44.4) | 27 (41.5) | 33 (47.1) |  |
| > 1 | 16 (11.9) | 7 (10.8) | 9 (12.9) |  |
| Bristol stool scale |  |  |  | 0.059 |
| Type 1 - severe constipation | 22 (15.6) | 17 (24.3) | 5 (7.0) |  |
| Type 2 - mild constipation | 36 (25.5) | 15 (21.4) | 21 (29.6) |  |
| Type 3,4 - normal | 70 (49.6) | 32 (45.7) | 38 (53.5) |  |
| Type 5 - lacking fiber | 9 (6.4) | 4 (5.7) | 5 (55.6) |  |
| Type 6 - mild diarrhea | 3 (2.1) | 4 (5.7) | 2 (2.8) |  |
| Type 7 - severe diarrhea | 1 (0.7) | 1 (1.4) | 0 (0.0) |  |

Values are mean (SD) or numbers (percentage). **Bold** style means statistical significance (*p* < 0.05).

*p*, comparison between the DA-9701 and placebo groups by ^a^ Student's t-test or ^b^ Fisher’s exact test.

Abbreviations: NDI-K = Nepean dyspepsia index-Korean version; QoL = quality of life; GI = gastrointestinal.

**Bold** style means statistical significance. *P* < 0.05 is significant for total NDI-K symptom score, dyspepsia score, and QoL scores, and for each item score significant *p*-values are shown as **Bold** applying for Bonferroni correction (15 items and 5 domains).

**Supplementary Table 2. Gastrointestinal symptoms and related quality of life change after intervention (Per Protocol set analysis)**

| Least Squares means from Linear mixed effects model | DA-9701 | | | | Placebo | | | | *p* -values | | | | |
| --- | --- | --- | --- | --- | --- | --- | --- | --- | --- | --- | --- | --- | --- |
|  | at 4 weeks | | at 12 weeks | | at 4 weeks | | at 12 weeks | | Between group | DA-9701 | | Placebo | |
|  | Mean change | std err | Mean change | std err | Mean change | std err | Mean change | std err | at 4 weeks | at 4 weeks | at 12 weeks | at 4 weeks | at 12 weeks |
| NDI-K symptom scale |  |  |  |  |  |  |  |  |  |  |  |  |  |
| Total symptom score | -5.24 | 1.55 | -6.80 | 2.27 | -2.20 | 1.91 | -3.19 | 1.29 | 0.219 | **0.001** | **0.003** | 0.252 | **0.014** |
| Dyspepia sum score (sum of 8 items) | -3.80 | 1.28 | -4.05 | 1.66 | -1.38 | 1.32 | -1.86 | 0.87 | 0.189 | **0.003** | **0.016** | 0.295 | **0.034** |
| NDI-K symptom items (Post-hoc analysis) |  |  |  |  |  |  |  |  |  |  |  |  |  |
| Pain in upper abdomen | -0.89 | 0.40 | -0.59 | 0.35 | -0.53 | 0.28 | -0.48 | 0.25 | 0.462 | 0.027 | 0.092 | 0.057 | 0.062 |
| Discomfort in upper abdomen | -0.64 | 0.35 | -0.58 | 0.34 | -0.45 | 0.30 | -0.43 | 0.25 | 0.679 | 0.071 | 0.088 | 0.137 | 0.081 |
| Burning in upper abdomen | -0.51 | 0.21 | -0.29 | 0.38 | -0.01 | 0.29 | 0.00 | 0.23 | 0.167 | 0.016 | 0.444 | 0.971 | 0.984 |
| Heartburn | 0.16 | 0.23 | -0.11 | 0.30 | 0.08 | 0.18 | 0.11 | 0.24 | 0.765 | 0.482 | 0.712 | 0.675 | 0.649 |
| Cramps in upper abdomen | 0.07 | 0.11 | 0.02 | 0.19 | -0.10 | 0.17 | 0.07 | 0.09 | 0.381 | 0.515 | 0.927 | 0.534 | 0.404 |
| Chest pain | -0.25 | 0.18 | -0.33 | 0.27 | -0.01 | 0.13 | -0.05 | 0.24 | 0.273 | 0.151 | 0.231 | 0.917 | 0.829 |
| Inability to finish regular meal | -0.51 | 0.37 | -0.38 | 0.45 | -0.38 | 0.31 | -0.61 | 0.35 | 0.788 | 0.168 | 0.402 | 0.222 | 0.084 |
| Bitter tasting fluid that comes to your mouth | -0.80 | 0.26 | -0.96 | 0.30 | -0.29 | 0.29 | -0.33 | 0.28 | 0.192 | **0.002** | **0.002** | 0.320 | 0.236 |
| Fullness after eating | -0.55 | 0.36 | -0.18 | 0.42 | 0.25 | 0.31 | -0.09 | 0.33 | 0.096 | 0.131 | 0.665 | 0.423 | 0.780 |
| Pressure in upper abdomen | 0.29 | 0.23 | -0.28 | 0.26 | 0.11 | 0.14 | 0.21 | 0.11 | 0.491 | 0.203 | 0.282 | 0.435 | 0.062 |
| Bloating in upper abdomen | -0.18 | 0.33 | -0.27 | 0.37 | 0.20 | 0.33 | 0.07 | 0.24 | 0.415 | 0.583 | 0.460 | 0.546 | 0.758 |
| Nausea | -0.82 | 0.36 | -1.44 | 0.42 | -0.57 | 0.29 | -0.54 | 0.22 | 0.594 | 0.025 | **0.001** | 0.052 | 0.017 |
| Belching | -0.49 | 0.31 | -0.73 | 0.36 | -0.41 | 0.32 | -0.63 | 0.21 | 0.861 | 0.119 | 0.042 | 0.199 | **0.003** |
| Vomiting | -0.20 | 0.32 | -0.56 | 0.28 | 0.09 | 0.14 | -0.13 | 0.10 | 0.410 | 0.535 | 0.049 | 0.529 | 0.173 |
| Bad breath | 0.07 | 0.13 | -0.07 | 0.25 | -0.17 | 0.29 | -0.29 | 0.23 | 0.441 | 0.571 | 0.765 | 0.553 | 0.221 |
| NDI-K QoL scale |  |  |  |  |  |  |  |  |  |  |  |  |  |
| Total QoL score | 6.57 | 1.74 | 8.85 | 1.80 | -0.82 | 2.26 | 2.03 | 2.68 | **0.010** | **<0.001** | **<0.001** | 0.716 | 0.450 |
| NDI-K QoL domains (Post-hoc analysis) |  |  |  |  |  |  |  |  |  |  |  |  |  |
| Tension/Sleep | 5.00 | 1.96 | 8.96 | 2.16 | -1.15 | 2.24 | -0.32 | 2.57 | 0.040 | 0.012 | **<0.001** | 0.608 | 0.901 |
| Interference with daily activities | 7.42 | 2.31 | 9.06 | 2.58 | 0.38 | 2.36 | 3.31 | 2.62 | 0.035 | **0.002** | **0.001** | 0.871 | 0.208 |
| Eating/Drinking | 7.78 | 2.59 | 9.25 | 2.90 | 0.52 | 2.67 | 2.04 | 2.69 | 0.053 | **0.003** | **0.002** | 0.845 | 0.448 |
| Knowledge/Control | 6.33 | 1.83 | 8.58 | 2.08 | 1.35 | 2.81 | 3.31 | 2.66 | 0.139 | **0.001** | **<0.001** | 0.631 | 0.214 |
| Work/Study | 6.34 | 2.41 | 8.56 | 2.20 | -5.26 | 4.29 | 1.85 | 6.96 | 0.019 | **0.009** | **<0.001** | 0.222 | 0.791 |

^*^Adjusted *p*-values for age, sex, baseline gastrointestinal symptom severity scores for each item.

Group and temporal changes in each score were analyzed by linear mixed effect models (See Methods in the Text)

Abbreviations: NDI-K = The Nepean dyspepsia index-Korean version; QoL = quality of life.

**Bold** style means statistical significance. *P* < 0.05 is significant for total NDI-K symptom score, dyspepsia score, and QoL scores, and for each item score significant *p*-values are shown as **Bold** applying for Bonferroni correction (15 items and 5 domains).

**Supplementary Table 3A. Gastrointestinal symptoms and related quality of life change after intervention in a subpopulation with the NDI-K total score >15**

| Least Squares means from Linear mixed effects model | DA-9701 | | | | Placebo | | | | *p* -values^*^ | | | | |
| --- | --- | --- | --- | --- | --- | --- | --- | --- | --- | --- | --- | --- | --- |
|  | at 4 weeks | | at 12 weeks | | at 4 weeks | | at 12 weeks | | Between group | DA-9701 | | Placebo | |
|  | Mean change | std err | Mean change | std err | Mean change | std err | Mean change | std err | at 4 weeks | at 4 weeks | at 12 weeks | at 4 weeks | at 12 weeks |
| NDI-K symptom scores |  |  |  |  |  |  |  |  |  |  |  |  |  |
| Total symptom score | -7.52 | 2.14 | -9.70 | 3.47 | -8.74 | 3.83 | -6.29 | 3.09 | 0.778 | **0.001** | **0.006** | **0.024** | **0.044** |
| Dyspepia sum score (sum of 8 items) | -5.77 | 1.69 | -5.84 | 2.55 | -4.86 | 2.76 | -4.15 | 1.82 | 0.780 | **0.001** | **0.024** | 0.081 | **0.024** |
| NDI-K symptom items (Post-hoc analysis) |  |  |  |  |  |  |  |  |  |  |  |  |  |
| Pain in upper abdomen | -1.36 | 0.58 | -0.87 | 0.55 | -0.98 | 0.53 | -0.68 | 0.51 | 0.632 | 0.022 | 0.118 | 0.065 | 0.186 |
| Discomfort in upper abdomen | -0.96 | 0.52 | -0.96 | 0.50 | -1.20 | 0.60 | -1.13 | 0.46 | 0.765 | 0.068 | 0.056 | 0.047 | 0.016 |
| Burning in upper abdomen | -0.62 | 0.29 | -0.58 | 0.56 | -0.68 | 0.55 | -0.64 | 0.42 | 0.918 | 0.035 | 0.301 | 0.217 | 0.127 |
| Heartburn | 0.34 | 0.36 | -0.13 | 0.48 | -0.36 | 0.24 | 0.02 | 0.58 | 0.119 | 0.350 | 0.793 | 0.141 | 0.973 |
| Cramps in upper abdomen | 0.03 | 0.18 | 0.03 | 0.30 | -0.42 | 0.38 | 0.02 | 0.20 | 0.287 | 0.877 | 0.918 | 0.262 | 0.909 |
| Chest pain | -0.19 | 0.24 | -0.28 | 0.41 | -0.04 | 0.09 | 0.31 | 0.52 | 0.565 | 0.426 | 0.495 | 0.614 | 0.553 |
| Inability to finish regular meal | -0.56 | 0.46 | -0.29 | 0.64 | -0.24 | 0.34 | -0.35 | 0.52 | 0.587 | 0.230 | 0.648 | 0.486 | 0.500 |
| Bitter tasting fluid that comes to your mouth | -1.01 | 0.38 | -1.32 | 0.44 | -0.37 | 0.51 | -0.38 | 0.45 | 0.318 | 0.009 | **0.003** | 0.468 | 0.406 |
| Fullness after eating | -1.00 | 0.46 | -0.06 | 0.60 | -0.74 | 0.55 | -0.82 | 0.60 | 0.719 | 0.031 | 0.920 | 0.181 | 0.176 |
| Pressure in upper abdomen | 0.37 | 0.33 | -0.56 | 0.38 | -0.13 | 0.25 | 0.31 | 0.23 | 0.237 | 0.271 | 0.141 | 0.605 | 0.184 |
| Bloating in upper abdomen | -0.50 | 0.45 | -0.55 | 0.52 | 0.39 | 0.58 | 0.30 | 0.40 | 0.225 | 0.267 | 0.298 | 0.497 | 0.455 |
| Nausea | -1.11 | 0.51 | -1.91 | 0.59 | -1.31 | 0.58 | -0.98 | 0.49 | 0.794 | 0.032 | **0.002** | 0.024 | 0.049 |
| Belching | -0.80 | 0.41 | -1.00 | 0.54 | -1.26 | 0.53 | -0.82 | 0.41 | 0.490 | 0.057 | 0.065 | 0.019 | 0.044 |
| Vomiting | -0.38 | 0.48 | -0.95 | 0.43 | -0.20 | 0.20 | -0.40 | 0.23 | 0.742 | 0.432 | 0.029 | 0.314 | 0.082 |
| Bad breath | 0.22 | 0.17 | -0.03 | 0.39 | -1.23 | 0.55 | -0.88 | 0.57 | 0.012 | 0.199 | 0.933 | 0.026 | 0.125 |
| NDI-K QoL scale |  |  |  |  |  |  |  |  |  |  |  |  |  |
| Total QoL score | 6.98 | 2.15 | 8.33 | 2.15 | 0.87 | 1.35 | 4.61 | 3.70 | **0.019** | **0.002** | **<0.001** | 0.522 | 0.215 |
| NDI-K QoL domains (Post-hoc analysis) |  |  |  |  |  |  |  |  |  |  |  |  |  |
| Tension/Sleep | 6.91 | 2.71 | 8.07 | 2.90 | -1.41 | 2.39 | -0.25 | 2.44 | 0.024 | 0.012 | **0.006** | 0.556 | 0.919 |
| Interference with daily activities | 8.92 | 2.98 | 9.41 | 2.90 | 0.81 | 1.85 | 5.03 | 2.59 | 0.025 | **0.003** | **0.002** | 0.663 | 0.054 |
| Eating/Drinking | 7.19 | 3.39 | 6.85 | 4.08 | 3.66 | 2.12 | -0.21 | 2.16 | 0.370 | 0.036 | 0.096 | 0.087 | 0.922 |
| Knowledge/Control | 4.44 | 2.23 | 8.58 | 2.58 | 2.37 | 1.81 | 3.57 | 2.53 | 0.474 | 0.048 | **0.001** | 0.191 | 0.161 |
| Work/Study | 7.03 | 2.88 | 8.04 | 2.60 | -1.10 | 2.02 | 15.13 | 13.59 | 0.023 | 0.016 | **0.002** | 0.587 | 0.268 |

^*^Adjusted *p*-values for age, sex, baseline gastrointestinal symptom severity scores for each item.

Group and temporal changes in each score were analyzed by linear mixed effect models (See Methods in the Text)

Abbreviations: NDI-K = the Nepean dyspepsia index-Korean version; QoL = quality of life.

**Bold** style means statistical significance. *P* < 0.05 is significant for total NDI-K symptom score, dyspepsia score, and QoL scores, and for each item score significant *p*-values are shown as **Bold** applying for Bonferroni correction (15 items and 5 domains).

**Supplementary Table 3B. Gastrointestinal symptoms and related quality of life change after intervention in a subpopulation with the NDI-K total score ≤15**

| Least Squares means from Linear mixed effects model | DA-9701 | | | | Placebo | | | | *p* -values | | | | |
| --- | --- | --- | --- | --- | --- | --- | --- | --- | --- | --- | --- | --- | --- |
|  | at 4 weeks | | at 12 weeks | | at 4 weeks | | at 12 weeks | | Between group | DA-9701 | | Placebo | |
|  | Mean change | std err | Mean change | std err | Mean change | std err | Mean change | std err | at 4 weeks | at 4 weeks | at 12 weeks | at 4 weeks | at 12 weeks |
| NDI-K symptom scale |  |  |  |  |  |  |  |  |  |  |  |  |  |
| Total symptom score | 3.74 | 2.31 | -1.97 | 1.07 | 1.71 | 1.17 | -1.11 | 0.77 | 0.437 | 0.108 | 0.070 | 0.148 | 0.152 |
| Dyspepia sum score (sum of 8 items) | 2.87 | 1.79 | -0.81 | 1.01 | 0.85 | 0.74 | -0.33 | 0.73 | 0.300 | 0.112 | 0.426 | 0.255 | 0.654 |
| NDI-K symptom items (Post-hoc analysis) |  |  |  |  |  |  |  |  |  |  |  |  |  |
| Pain in upper abdomen | -0.20 | 0.14 | -0.21 | 0.16 | -0.17 | 0.22 | -0.26 | 0.22 | 0.936 | 0.169 | 0.177 | 0.425 | 0.247 |
| Discomfort in upper abdomen | 0.63 | 0.36 | 0.13 | 0.27 | 0.14 | 0.21 | 0.08 | 0.22 | 0.245 | 0.085 | 0.639 | 0.507 | 0.707 |
| Burning in upper abdomen | -0.35 | 0.33 | 0.12 | 0.34 | 0.39 | 0.23 | 0.41 | 0.22 | 0.069 | 0.296 | 0.721 | 0.094 | 0.067 |
| Heartburn | 0.42 | 0.33 | 0.00 | 0.01 | 0.27 | 0.21 | 0.09 | 0.09 | 0.694 | 0.202 | 0.731 | 0.191 | 0.318 |
| Cramps in upper abdomen | 0.00 | 0.00 | 0.00 | 0.01 | 0.07 | 0.07 | 0.09 | 0.09 | 0.300 | 0.565 | 0.729 | 0.309 | 0.302 |
| Chest pain | -0.20 | 0.14 | -0.21 | 0.16 | 0.01 | 0.17 | -0.23 | 0.16 | 0.362 | 0.164 | 0.187 | 0.957 | 0.153 |
| Inability to finish regular meal | -0.18 | 0.48 | -0.55 | 0.51 | -0.63 | 0.44 | -0.84 | 0.43 | 0.496 | 0.701 | 0.284 | 0.150 | 0.053 |
| Bitter tasting fluid that comes to your mouth | 0.35 | 0.39 | -0.25 | 0.20 | -0.27 | 0.27 | -0.31 | 0.31 | 0.194 | 0.367 | 0.206 | 0.330 | 0.320 |
| Fullness after eating | 1.31 | 0.60 | -0.10 | 0.39 | 0.68 | 0.28 | 0.35 | 0.32 | 0.347 | 0.032 | 0.807 | 0.016 | 0.280 |
| Pressure in upper abdomen | 0.54 | 0.24 | 0.26 | 0.17 | 0.22 | 0.12 | 0.17 | 0.12 | 0.245 | 0.028 | 0.117 | 0.076 | 0.166 |
| Bloating in upper abdomen | 1.10 | 0.45 | 0.14 | 0.42 | 0.22 | 0.34 | 0.03 | 0.28 | 0.116 | 0.015 | 0.736 | 0.518 | 0.904 |
| Nausea | -0.04 | 0.51 | -0.73 | 0.45 | 0.06 | 0.22 | -0.22 | 0.13 | 0.864 | 0.940 | 0.107 | 0.802 | 0.086 |
| Belching | 0.13 | 0.23 | -0.25 | 0.14 | 0.14 | 0.29 | -0.40 | 0.20 | 0.977 | 0.582 | 0.076 | 0.636 | 0.041 |
| Vomiting | 0.25 | 0.25 | 0.01 | 0.01 | 0.22 | 0.16 | 0.00 | 0.00 | 0.905 | 0.310 | 0.519 | 0.174 | 0.489 |
| Bad breath | -0.10 | 0.10 | -0.11 | 0.11 | 0.44 | 0.21 | 0.06 | 0.06 | 0.020 | 0.318 | 0.313 | 0.036 | 0.296 |
| NDI-K QoL scale |  |  |  |  |  |  |  |  |  |  |  |  |  |
| Total QoL score | 2.98 | 3.49 | 8.41 | 3.13 | -2.75 | 3.09 | -0.19 | 3.42 | 0.221 | 0.394 | **0.008** | 0.374 | 0.955 |
| NDI-K QoL domains (Post-hoc analysis) |  |  |  |  |  |  |  |  |  |  |  |  |  |
| Tension/Sleep | 1.73 | 3.45 | 9.70 | 3.16 | -1.81 | 2.86 | -1.20 | 3.66 | 0.432 | 0.617 | **0.003** | 0.529 | 0.743 |
| Interference with daily activities | 2.23 | 3.62 | 7.56 | 4.63 | -1.43 | 3.22 | 1.49 | 3.77 | 0.453 | 0.539 | 0.105 | 0.659 | 0.693 |
| Eating/Drinking | 5.77 | 4.28 | 10.88 | 3.65 | -2.60 | 3.62 | 3.17 | 3.94 | 0.138 | 0.180 | **0.003** | 0.473 | 0.422 |
| Knowledge/Control | 3.03 | 3.88 | 6.50 | 3.38 | -0.35 | 3.80 | 2.40 | 3.77 | 0.536 | 0.437 | 0.057 | 0.927 | 0.527 |
| Work/Study | 2.45 | 3.80 | 7.75 | 3.83 | -7.49 | 5.67 | -6.87 | 6.28 | 0.152 | 0.521 | 0.045 | 0.189 | 0.276 |

^*^Adjusted *p*-values for age, sex, baseline gastrointestinal symptom severity scores for each item.

Group and temporal changes in each score were analyzed by linear mixed effect models (See Methods in the Text)

Abbreviations: NDI-K = the Nepean dyspepsia Index-Korean version; QoL = quality of life.

**Bold** style means statistical significance. *P* < 0.05 is significant for total NDI-K symptom score, dyspepsia score, and QoL scores, and for each item score significant *p*-values are shown as **Bold** applying for Bonferroni correction (15 items and 5 domains).

**Supplementary Table 4. Gastrointestinal symptoms diary changes after 4 and 12 weeks**

|  | DA-9701-first treated group | | | |  | | Placebo-first treated group | | | |  |  | | |  |  |  |
| --- | --- | --- | --- | --- | --- | --- | --- | --- | --- | --- | --- | --- | --- | --- | --- | --- | --- |
|  | Baseline | At 4 weeks | At 12 weeks | *P_4_^a^* | | *P_12_^a^* | | Baseline | At 4 weeks | At 12 weeks | | | *P_4_^a^* | *P_12_^a^* | | | |
| Inability to finish regular meal due to early fullness/day |  |  |  | 0.118 | | 0.101 | |  |  |  | | | 0.180 | **0.031** | | | |
| Absent | 34 (50.0) | 39 (61.9) | 30 (57.7) |  | |  | | 45 (62.5) | 41 (64.1) | 42 (77.8) | | |  |  | | | |
| Once | 17 (25.0) | 8 (12.7) | 11 (21.2) |  | |  | | 15 (20.8) | 18 (28.1) | 10 (18.5) | | |  |  | | | |
| Twice | 4 (5.9) | 4 (6.3) | 3 (5.8) |  | |  | | 6 (8.3) | 2 (3.1) | 1 (1.9) | | |  |  | | | |
| Three times (every meal) | 13 (19.1) | 12 (19.0) | 8 (15.4) |  | |  | | 6 (8.3) | 3 (4.7) | 1 (1.9) | | |  |  | | | |
| Bloating in upper abdomen after meal/day |  |  |  | 0.927 | | 0.210 | |  |  |  | | | 0.529 | 0.283 | | | |
| Absent | 40 (58.8) | 39 (60.9) | 34 (65.4) |  | |  | | 47 (65.3) | 47 (73.4) | 38 (69.1) | | |  |  | | | |
| Once | 17 (25.0) | 12 (18.8) | 11 (21.2) |  | |  | | 19 (26.4) | 11 (17.2) | 16 (29.1) | | |  |  | | | |
| Twice | 5 (7.4) | 7 (10.9) | 4 (7.7) |  | |  | | 3 (4.2) | 3 (4.7) | 1 (1.8) | | |  |  | | | |
| Three times (every meal) | 6 (8.8) | 6 (9.4) | 3 (5.8) |  | |  | | 3 (4.2) | 3 (4.7) | 0 (0.0) | | |  |  | | | |
| Burning or pain in upper abdomen/day |  |  |  | **0.029** | | 0.186 | |  |  |  | | | 0.216 | 0.912 | | | |
| Absent | 46 (66.7) | 50 (79.4) | 40 (76.9) |  | |  | | 54 (75.0) | 53 (84.1) | 47 (85.5) | | |  |  | | | |
| Mild | 19 (27.5) | 11 (17.5) | 8 (15.4) |  | |  | | 15 (20.8) | 9 (14.3) | 4 (7.3) | | |  |  | | | |
| Moderate | 1 (1.4) | 1 (1.6) | 3 (5.8) |  | |  | | 2 (2.8) | 1 (1.6) | 1 (1.8) | | |  |  | | | |
| Severe | 2 (2.9) | 0 (0.0) | 1 (1.9) |  | |  | | 1 (1.4) | 0 (0.0) | 2 (3.6) | | |  |  | | | |
| Very severe | 1 (1.4) | 1 (1.6) | 0 (0.0) |  | |  | | 0 (0.0) | 0 (0.0) | 1 (1.8) | | |  |  | | | |
| Defecation frequency/day |  |  |  | >0.999 | | >0.999 | |  |  |  | | | 0.655 | 0.366 | | | |
| < 1 | 31 (47.7) | 32 (50.0) | 27 (51.9) |  | |  | | 28 (40.0) | 29 (46.0) | 23 (42.6) | | |  |  | | | |
| 1 | 27 (41.5) | 23 (35.9) | 17 (32.7) |  | |  | | 33 (47.1) | 24 (38.1) | 25 (46.3) | | |  |  | | | |
| > 1 | 7 (10.8) | 9 (14.1) | 8 (15.4) |  | |  | | 9 (12.9) | 10 (15.9) | 6 (11.1) | | |  |  | | | |
| Bristol stool scale |  |  |  | 0.329 | | 0.315 | |  |  |  | | | 0.914 | 0.478 | | | |
| Type 1 - severe constipation | 17 (24.3) | 13 (20.3) | 6 (11.5) |  | |  | | 5 (7.0) | 3 (4.7) | 3 (5.5) | | |  |  | | | |
| Type 2 - mild constipation | 15 (21.4) | 21 (32.8) | 16 (30.8) |  | |  | | 21 (29.6) | 20 (31.3) | 20 (36.4) | | |  |  | | | |
| Type 3,4 - normal | 32 (45.7) | 28 (43.8) | 24 (46.2) |  | |  | | 38 (53.5) | 35 (54.7) | 24 (43.6) | | |  |  | | | |
| Type 5 - lacking fiber | 4 (5.7) | 1 (1.6) | 3 (5.8) |  | |  | | 5 (7.0) | 3 (4.7) | 5 (9.1) | | |  |  | | | |
| Type 6 - mild diarrhea | 1 (1.4) | 1 (1.6) | 1 (1.9) |  | |  | | 2 (2.8) | 3 (4.7) | 3 (5.5) | | |  |  | | | |
| Type 7 - severe diarrhea | 1 (1.4) | 0 (0.0) | 2 (3.8) |  | |  | | 0 (0.0) | 0 (0.0) | 0 (0.0) | | |  |  | | | |

Values are numbers (percentage).

Bold style means statistical significance (*p* < 0.05).

*P_4_^a^*, Comparison between baseline and after 4 weeks in each group by Wilcoxon singed rank test.

*P_12_^a^,* Comparison between baseline and after 12 weeks in each group by Wilcoxon singed rank test.

**Supplementary Table 5. Parkinsonian symptoms and PD-related quality of life change after intervention**

|  | DA-9701 | | | | Placebo | | | | *p* -values^*^ | | | | |
| --- | --- | --- | --- | --- | --- | --- | --- | --- | --- | --- | --- | --- | --- |
|  | at 4 weeks | | at 12 weeks | | at 4 weeks | | at 12 weeks | | Between group | DA-9701 | | Placebo | |
|  | Mean change | std err | Mean change | std err | Mean change | std err | Mean change | std err | at 4 weeks | at 4 weeks | at 12 weeks | at 4 weeks | at 12 weeks |
| UPDRS part III score^†^ | -0.42 | 0.63 | -1.87 | 1.12 | -0.94 | 0.59 | -0.75 | 0.91 | 0.557 | 0.502 | 0.096 | 0.114 | 0.411 |
| K-PDQ39 Summary Index^†^ | 0.22 | 1.25 | -0.91 | 1.28 | 0.24 | 1.09 | 0.37 | 1.10 | 0.991 | 0.859 | 0.478 | 0.825 | 0.737 |
| K-PDQ39 domains |  |  |  |  |  |  |  |  |  |  |  |  |  |
| Mobility | 0.54 | 1.56 | 1.16 | 1.94 | 1.16 | 1.69 | 0.77 | 1.76 | 0.789 | 0.728 | 0.549 | 0.493 | 0.662 |
| Activities of daily living | 1.33 | 1.93 | 0.47 | 1.66 | 0.35 | 1.08 | 1.67 | 1.70 | 0.657 | 0.494 | 0.778 | 0.748 | 0.328 |
| Emotional well-being | -0.15 | 2.38 | -2.05 | 2.05 | -0.14 | 1.36 | -0.89 | 1.22 | 0.995 | 0.948 | 0.319 | 0.919 | 0.465 |
| Stigma | 0.28 | 1.51 | -2.19 | 1.50 | 1.12 | 1.94 | 0.89 | 2.17 | 0.733 | 0.851 | 0.146 | 0.563 | 0.682 |
| Social support | -0.83 | 1.61 | -1.81 | 1.84 | -0.35 | 1.72 | -0.15 | 1.78 | 0.838 | 0.604 | 0.325 | 0.839 | 0.931 |
| Cognition | 1.25 | 1.77 | 1.72 | 1.94 | 0.11 | 1.52 | 2.30 | 2.00 | 0.627 | 0.482 | 0.374 | 0.944 | 0.253 |
| Communication | 0.47 | 1.86 | 1.71 | 2.09 | -0.28 | 1.72 | 1.47 | 1.68 | 0.768 | 0.802 | 0.414 | 0.870 | 0.383 |
| Bodily discomfort | -1.03 | 1.91 | -5.43 | 2.48 | 0.53 | 1.68 | -3.23 | 1.95 | 0.542 | 0.590 | 0.030 | 0.755 | 0.098 |

^*^Adjusted *p*-values for age, sex, baseline scores for each item.

^†^significant if *p* < 0.05.

For each item score, the mean change is significant if *p* < 0.00625 by Bonferroni correction for multiple comparison.

Group and temporal changes in each score were analyzed by linear mixed effect models (See Methods in the Text)

Abbreviations: UPDRS = The Unified Parkinson’s Disease Rating Scale; K-PDQ39= the Korean version 39-item PD Questionnaire.

**Supplementary Table 6.** **Adverse drug reactions in this trial**

|  | First 4 weeks’ intervention | | | Additional 8 weeks’ DA-9701 treatment | Safety follow-up  (+ 2 weeks) |
| --- | --- | --- | --- | --- | --- |
|  | DA-9701 group | Placebo group | *p* |  |  |
| Total adverse drug reaction | 10 (15.4) | 5 (7.7) | 0.173 | 3 (2.7) | 1 (0.9) |
| List of adverse drug reaction |  |  |  |  |  |
| Abdominal discomfort | 1 (1.5) | 0 (0.0) |  | 2 (1.8) |  |
| Constipation | 2 (3.1) | 0 (0.0) |  |  |  |
| Inclination for stool | 0 (0.0) | 1 (1.5) |  |  |  |
| Diarrhea | 0 (0.0) | 1 (1.5) |  |  |  |
| Nausea | 1 (1.5) | 0 (0.0) |  |  |  |
| Sleep disturbance | 2 (3.1) | 1 (1.5) |  |  |  |
| Dizziness | 2 (3.1) | 1 (1.5) |  |  |  |
| Dizziness with headache | 0 (0.0) | 1 (1.5) |  |  |  |
| Lethargy with slowness of movement | 1 (1.5) | 0 (0.0) |  |  |  |
| Skin rash | 1 (1.5) | 0 (0.0) |  |  |  |
| Orolingual dyskinesia |  |  |  | 1 (0.9) |  |
| Tinnitus |  |  |  |  | 1 (0.9) |
| Aggravation of aggressiveness |  |  |  | 1 (0.9) |  |
| Aggravation of restless legs syndrome |  |  |  | 1 (0.9) |  |

Values are numbers (percentage). *p*, comparison between the DA-9701 and placebo groups by Chi-squared test.
