# Supplementary figures and images for: Double‐Blind, Randomized, Placebo‐Controlled Trial of DA‐9701 in Parkinson's Disease: PASS‐GI Study
Source: Mov Disord. 2020 Aug 6;35(11):1966–76. doi: 10.1002/mds.28219 (PMC7754502; doi:10.1002/mds.28219)

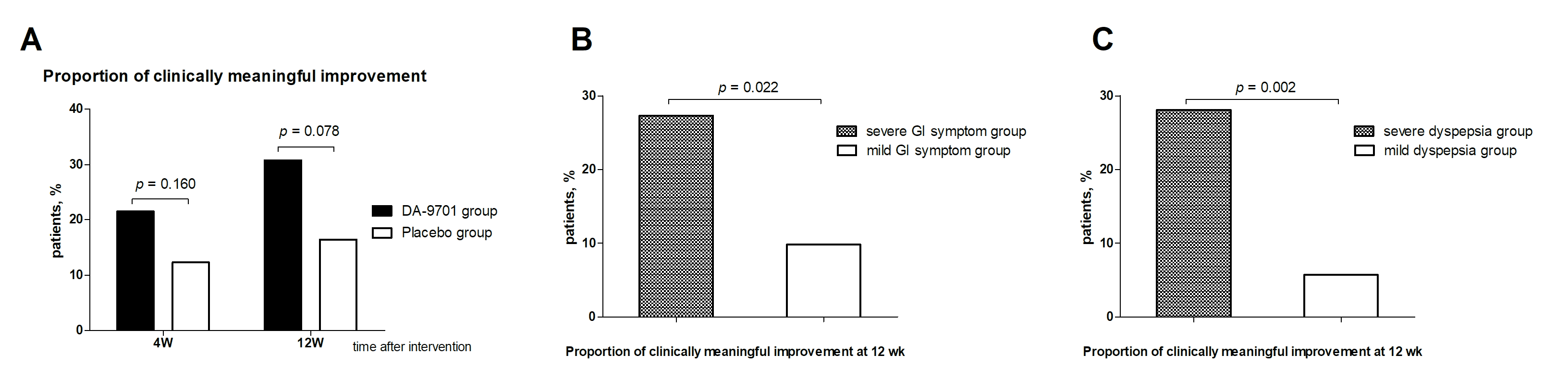

Supplement: Supplementary file 2 — Supplementary figure 1 Proportion of clinically meaningful improvement after DA‐9701 therapy in study participants. (A) The proportion of patients with clinically meaningful improvement (≥50% reduction in the score from baseline) between DA‐9701 and the placebo groups at 4 and 12 weeks of intervention. (B‐C) Comparison of proportion of clinically meaningful improvement at 12 weeks between patients with severe gastrointestinal symptoms and those with mild ones at baseline. Comparisons by the NDI‐K symptom total score > 15 or not (B) and by the dyspepsia sum score > 10 or not (C). Abbreviations: NDI‐K = The Nepian Dyspepsia Index‐Korean version [file MDS-35-1966-s002.tif]
